# Perioperative Fasting and the Patient Experience

**DOI:** 10.7759/cureus.1272

**Published:** 2017-05-24

**Authors:** Telliane Chon, Alfred Ma, Connie Mun-Price

**Affiliations:** 1 Anesthesiology, Riverside University Health System, Moreno Valley, California, United States

**Keywords:** preop fasting, patient experience, surgery

## Abstract

Standard preparation for a surgical procedure requires patients to fast (nulla per os [NPO]) after midnight before their operation. Unfortunately, given the unpredictable nature of operating room scheduling and unavoidable delays, patients may find themselves anxiously waiting and fasting much longer than expected. In recent years, the usefulness of prolonged fasting to prevent pulmonary aspiration has been questioned. According to the American Society of Anesthesiologists (ASA) guidelines, unnecessarily prolonged fasting can be avoided by allowing patients to have clear liquids with the minimal fasting time of only two hours. This study examines a random sampling of elective scheduled surgeries at a 439-bed safety-net teaching hospital in Southern California in October 2016. The study revealed significantly prolonged NPO times caused by delays in the scheduling of operation times. An analysis of delays revealed that prior surgical procedures running longer than scheduled were the most common reason for a delay in starting an operation and, subsequently, prolonging patient fasting time. Significantly prolonged fasting times warrant the need for institutional management strategy changes and a revamping of clinical education curriculums.

## Introduction

Delays to scheduled operations occur frequently in most busy operating rooms [1]. This may result in patient frustration, anxiety, and anger. Standard preparation for many surgical procedures requires patients to fast [2]. This is to avoid the possibility of pulmonary aspiration of the stomach contents during the operation. Unfortunately, given the unpredictable nature of an operating room and unavoidable delays, patients may find themselves waiting and fasting longer than expected on the day of the operation. In some instances, scheduled operations may be canceled in the late afternoon, necessitating rescheduling and another round of presurgical fasting. These changes negatively impacted the experience of the patients and their families as their expectations were not met. This study was designed to investigate the occurrence of prolonged fasting times with the goal of finding ways to improve patients’ surgical experiences.

The first published guideline on preoperative fasting was released in 1883 by Joseph Lister, the British surgeon who founded antiseptic medicine [3]. He advised, “while it is desirable that there should be no solid matter in the stomach when chloroform is administered, it will be found very salutary to give a cup of tea or a beef-tea about two hours previously.” The modern practice of fasting prior to an operation began with Dr. Curtis Mendelson’s observations in 1946 [4]. He reported that surgical patients who ingested food shortly before their surgical procedure were more likely to regurgitate their stomach contents with severe consequences. The practice of fasting preoperatively is based on the premise that fasting allows time for gastric emptying to occur, thereby reducing the risk of aspiration pneumonitis during anesthesia.

Prolonged fasting is not without harm to patients, especially those with medical comorbidities. Prolonged fasting can raise the risk of an attack in people with gout [5]. Fasting can quickly result in dehydration and the need for fluid replacement during anesthesia in addition to increasing the potential for replacing blood lost during the surgical procedure [6]. Dehydration and blood loss during the surgical procedure reduces blood volume and alters drug kinetics, which can lead to more side effects [7].

In 2011, the American Society of Anesthesiologists (ASA) published an updated practice guideline addressing the practice of “nulla per os” (NPO) after midnight [8]. The purpose of the guideline was to enhance the safety of anesthesia care by reducing the risk of complications due to the pulmonary aspiration of gastric contents. Highlights from the 2011 guidelines include a risk assessment for identifying patients at a higher risk for aspiration and recommended techniques to mitigate these risks [8]. For example, low-risk patients are required to abstain from solid food for six to eight hours prior to surgery. Clear liquids are encouraged up to two hours before surgery [8]. Patients should be informed of fasting requirements (and the reasons for those requirements) sufficiently in advance of their procedures. Verification of patient compliance with fasting requirements should be assessed at the time of the procedures [8]. This paper postulates that the lack of enforcement of the ASA fasting guidelines may be a key factor in the unnecessary prolongation of fasting in today’s surgical practice.

## Materials and methods

The study was conducted in a safety-net hospital, Riverside County Regional Medical Center (RCRMC) in Moreno Valley, California. It is a 439-bed teaching hospital that is a level II trauma center providing both adult and pediatric trauma services. RCRMC is home to five different specialty residency programs consisting of anesthesiology, general surgery, orthopedics, internal medicine, and family medicine. The hospital also accommodates other specialty resident training rotations such as otolaryngology, urology, neurosurgery, obstetrics and gynecology, and ophthalmology from various programs in Southern California. RCRMC has nine operating rooms that utilize block scheduling, where individual surgical services are assigned to utilize several operating rooms during a certain day or days of the week. A master operating room schedule is then developed daily.

Through the McKesson Electronic Scheduling Data System at RCRMC, a sample of cases that involved resident training was collected from a pool of weekday operation schedules in October 2016. Data were subdivided into elective cases and urgent add-ons or emergency cases. Among the elective cases, the cases were further subdivided into first cases of the day and other subsequent elective cases. The study group data collection excluded the first cases in each operating room and the urgent add-ons or emergency cases. The goal was to observe more predictable operation scheduling and eliminate any potential outliers that would skew the numeric representation of NPO status during standard scheduling protocol and process. The study design is shown in Table 1.

**Table 1:** Study design chart

Total cases involve resident training during October 2016			
First cases of the day	Added-on cases	Other elective cases	
First cases, urgent and emergency cases		On time cases	Significantly delays cases
Excluded from the study		Included in the study	

After an elective surgical procedure is scheduled, on the day of the operation, a patient is transported to the pre-operating holding room 45 minutes prior to the available operation time. In the holding room, a preoperative surgical admission procedure is conducted according to protocols and established policies. When the operating suite is available and ready, the patient then is transported to the operating suite for anesthesia, and the operation then proceeds.

In this study, documented times were collected for the scheduled start times according to the daily operating room template (i.e., Scheduled Start Time), arrival at the holding room from the same day surgical procedure admission or inpatient room (i.e., Holding Room Time), the initiation of anesthesia (i.e., Anesthesia Start Time), and the calculated total NPO status. The total difference in minutes between “Scheduled Start time” and “Anesthesia Start time” was also calculated per surgical service. NPO status was based on the patient being without food or liquid oral consumption past midnight the day prior to the scheduled surgical procedure, as recommended by surgeons and anesthesiologists.

The flow of operating rooms and several definitions are provided to assist with data interpretation in Table 2.

**Table 2:** Data sampling definition NPO: nulla per os; OR: operating room.

Term	Definition
Scheduled Start Time	Time surgical case is scheduled to start based on daily OR template
Holding Room Time	Time patient has arrived in preoperative holding suite from Same Day Surgery admission or inpatient room
Anesthesia Start Time	Anesthesia provider assumes patient care
Difference	Minutes difference between Scheduled Start and Anesthesia Start
NPO	Minutes difference between Anesthesia Start and midnight the day prior to the surgical procedure

The sample of cases involving resident training was collected from operations of various sub-specialties: General Surgery, Otolaryngology, Ophthalmology, Gynecology, Orthopedics, Urology, and Neurosurgery surgical services.

Surgical delay status was evaluated in detail to establish the potential reasons that result in prolonging NPO practices in this study.

## Results

Five hundred eighteen cases involving resident training were performed during the month of October in 2016. Of those, 86 were classified as add-on cases. Add-on cases are urgent or emergency surgeries that were put onto the master schedule within 24 hours of the day of the operation. Given the time available, add-on cases were performed depending on the level of urgency or on a first come, first served basis. Together with the first cases of the day, add-on cases were excluded from the study. Among the 518 cases, 270 cases (51.2%) were included in the study based on the inclusion and exclusion criteria as indicated above.

Among the cases included in the study, 102 cases started at their scheduled times, and 168 cases (62.2%) were recorded as significantly delayed. Cases considered to be significantly delayed were cases that were delayed by 60 minutes or more from the operating room template scheduled time. The top six reasons for significant delays are identified in Table 3. A previous case taking longer than scheduled was the most frequent reason for case delay and prolonged patient fasting time, accounting for 59.5% of the delayed cases. The second most frequent reason for a delay was case order changes (14.3%). Many times, surgeons decide to alter the sequence of cases in the operating room on the day of operation. This resulted in delays because patients may not be ready or have not yet been admitted to the holding area. The third most frequent reason for a delay was provider tardiness (11%). This category can be due to providers conducting surgical procedures in another operating room, in the clinic, or the provider is simply not present in the surgery department.

**Table 3:** Criteria of delay NPO: nulla per os.

Criteria of Case Delay	Definition	Events
Previous Case Run Over	Previous case runs over resulted in delay of the case to follow by 60 minutes	100
Case Order Change	Provider manipulated case order on the day of surgical procedure resulted in delay of case schedule time over 60 minutes	24
Provider Late	Provider is not in the operating room 15 minutes after the scheduled time, resulted in case delayed over 60 minutes	18
Patient Late	Patient is not in the preoperative holding area 30 minutes prior to the scheduled time and resulted in case delay over 60 minutes	12
Bump by Emergency	Emergency case takes priority for availability of room and provider, resulted in original case delay over 60 minutes	5
Patient Not NPO	Patient arrived at preoperative holding without adequate NPO status and resulted in case delay over 60 minutes	3
Others	Medical clearance required, patient is not ready, etc.	6
TOTAL		168

The average NPO time from these cases (N = 270) was 769.8 ± 129.6 minutes. This average includes 102 cases that were performed on time according to the schedule template. Among the cases that were categorized as significantly delayed (N = 168), the average NPO time was 810.4 ± 104.8 minutes.

The average time of case delay was 152.2 ± 102.8 minutes from all included cases (N = 270). Those that were categorized as significantly delayed had an average time of 189.2 ± 91.9 minutes (N = 168). There was no statistical difference in comparing the two sets of data presented in Table 4.

**Table 4:** Average time of delay NPO: nulla per os.

	Case Included (N = 270)	Case Significantly Delayed (N = 168)
NPO Time (Minutes)	769.83 ± 129.58	810.43 ± 104.76
Case Delayed (Minutes)	152.15 ± 102.76	189.19 ± 91.89

Further, we compared the fasting time between the expected schedule time and the real time for when the operation took place. The discrepancy between the expected fasting time and real fasting time is more than three hours. The ASA fasting guideline for surgical procedures is eight hours, yet the average fasting time for patients having surgical procedures at RCRMC is more than 10.5 hours. The significantly delayed group often experienced even more delays by an average of three hours. This addition to the already prolonged fasting time resulted in a patient fasting time of nearly 14 hours.

## Discussion

Current surgical practices often demand that all patients abstain from all food or drink after midnight regardless of whether the operation is at 7:00 a.m. or 5:00 p.m. the following day. While it is important to adhere to accepted practice guidelines, this study suggests that patients are often kept too long without nutrition or fluids. In addition to discomfort, there are many medical conditions in which this scenario would cause harm to a patient.

The data from this study support the notion that NPO times are lengthened beyond recommended time frames. These findings also imply that patients’ well-being and comfort are not deemed an institutional priority, and this habit of patient care can hardly be considered good practice.

Too often, medical providers are reluctant to address NPO time management issues because they desire the convenience of case movement and case order change during the day of the operation [9]. In medical teaching facilities, delays frequently occur in the operating room and have a major effect on patient flow and, ultimately, resource utilization. Many times, cases are canceled and rescheduled because there is not enough time left in the day to finish the surgery. This raises the question of whether medical provider convenience outweighs patient stress and prolonged hunger. Management strategy processes often do not address these issues, which may occasionally result in significant dilemmas and adverse medical outcomes [10]. Better communication between medical providers as well as scheduling realistic procedure times can help resolve these delays in a teaching institution. This process may result in scheduling fewer procedures, but may also lead to an improvement in the patient experience.

## Conclusions

Having a hungry and thirsty patient anxiously waiting for a procedure adversely impacts the patient's experience. In a teaching hospital, learning is part of the mission of the organization, but overemphasizing teaching in such a healthcare organization may negatively impact the patient experience. Efficient utilization and management of the operating rooms and proper surgical scheduling are critical to running an effective operating room suite. Better surgical preparation of patients and effective management of patient NPO time as expected by the process can mitigate unnecessary complaints by unsatisfied patients and families.

Finally, the use of fasting status provides an accurate measure of the patient experience, patient safety, and operational efficiency. It is particularly valuable for objectively measuring the patient’s experience. For example, asking a general question of how a patient felt about his experience is an indirect measure of several variables and is ultimately subjective. Monitoring patients’ fasting times yields an objective look at safety, efficiency, and experience variables. Monitoring fasting times would also be valuable both internally to monitor and track progress and externally to compare institutions with each other.
